# Dalbavancin reduces biofilms of methicillin-resistant *Staphylococcus aureus* (MRSA) and methicillin-resistant *Staphylococcus epidermidis* (MRSE)

**DOI:** 10.1007/s10096-016-2845-z

**Published:** 2016-11-28

**Authors:** D. Knafl, S. Tobudic, S. C. Cheng, D. R. Bellamy, F. Thalhammer

**Affiliations:** 1 Division of Infectious Diseases and Tropical Medicine, Department of Medicine I, Medical University Vienna, Waehringer Guertel 18-20, 1090 Vienna, Austria; 2 Dunedin School of Medicine, University of Otago, Dunedin, New Zealand; 3 Dunedin School of Medicine, University of Otago, Wellington, New Zealand

## Abstract

Activity of dalbavancin against methicillin-resistant *Staphylococcus aureus* (MRSA) and methicillin-resistant *Staphylococcus epidermidis* (MRSE) in biofilm was investigated and the microbicidal biofilm concentrations (MBC) were determined. Biofilms obtained from ten MRSA and ten MRSE bloodstream isolates, collected from patients in the General Hospital of Vienna between 2012 and 2015, were incubated with dalbavancin in trypticase soy broth (TSB) in serial dilution from 0.0625 mg/l to 256 mg/l using a microtiter plate biofilm model. The plates were incubated for 24 h at 37 ° C and 50% humidity. Biofilms were fixed with 2.5% glutaraldehyde and stained with crystal violet. Subsequently the optical density (OD_620_) was used to measure the MBC, defined as the concentration of dalbavancin leading to a 50% reduction of biofilm. MBC for MRSA was 1 mg/l–4 mg/l (minimal inhibitory concentrations (MIC) 0.0312 mg/l–0.064 mg/l). MBC for MRSE was 2 mg/l–16 mg/l (MIC 0.023 mg/l–0.0625 mg/l). Dalbavancin successfully reduced MRSA and MRSE in biofilms, and therefore provides a promising option for the treatment of biofilm-associated infections.

## Introduction

Medical devices, such as intravenous catheters, prosthetic heart valves, and vascular or joint prostheses, have contributed to reducing morbidity and mortality for numerous patients every year [1]. However, they are fraught with the risk of leading to surface-associated infections through formation of bacterial or fungal biofilms [1]. Although explantation of the infected device is the preferred treatment option, in some cases removal is not feasible due to absolute necessity of the implant, difficulty of surgery, or inoperability of the patient, to mention only a few reasons. Another problem with bacterial biofilm infections is that they are often resistant to antibiotic therapy despite the sensitivity of the single planktonic organisms to the antibiotic [1, 2]. *Staphylococcus aureus* (*S. aureus*) and *Staphylococcus epidermidis* (*S. epidermidis*) are among the most common pathogens associated with surface-associated infections [1–3]. Unfortunately, methicillin-resistant *S. aureus* (MRSA) and methicillin-resistant *S. epidermidis* (MRSE) are also very prevalent nosocomial pathogens, often responsible for medical device infections [4, 5]. Dalbavancin is a novel semisynthetic lipoglycopeptide with unique pharmacokinetic properties allowing weekly dosing [6]. Recent data indicate that dalbavancin may have antimicrobial potential against prokaryotic biofilms [7, 8]. Furthermore, it has a broad antimicrobial gram-positive spectrum (>6,000 g-positive isolates) including activity against methicillin-resistant *staphylococci*, which show remarkable susceptibility to dalbavancin (e.g., MIC-range *S. aureus* ≤ 0.015–0.25 mg/l; MIC-range coagulase-negative *staphylococci* ≤ 0.015–0.25 mg/l) [9]. Its high efficacy paired with its dosage regimen provides the opportunity to treat patients with gram-positive infections, normally requiring inpatient antibiotics, as outpatients. The objective of this study was to investigate the in-vitro activity of dalbavancin against MRSA and MRSE isolates.

## Methods

In our study, we used ten MRSA and ten MRSE bloodstream isolates acquired from patients treated in the General Hospital of Vienna between 2012 and 2015. Preparation of sterile stock solutions of dalbavancin (Xydalba®, Durata Therapeutics International B.V. ®, Chicago, IL, USA) was performed in trypticase soy broth (TSB) (Oxoid®, Hampshire, UK). MICs were determined using the microdilution method and were confirmed with E-tests according to the procedures outlined by the Clinical and Laboratory Standards Institute (CLSI). A modified Christensen method was used for biofilm formation, with the strains grown overnight on Columbia-blood-agar (BioMerieux®, Marcy-l’Étoile, France) [10]. Twenty ml of TSB was then inoculated with a large loop of each strain and shaken at 90–100 rpm in a brood chamber at 36 °C and 50% humidity for 24 h. The bacterial suspensions were diluted 1:100 in TSB, equivalent to a colony count of 10^7^ CFU/ml, and incubated on a shaker with 90–100 rpm in the same brood chamber for 4 h. Then, 200 μl aliquots of bacterial solutions were placed into 84 wells of a 96-well polystyrene flat-bottomed microtiter plate (Cellstar®, greiner bio-one®, Frickenhausen, Germany). The remaining 12 wells served as negative TSB-only controls. After 24 h of incubation, media and planktonic cells were removed and 200 μl/well dalbavancin dissolved in TSB in serial dilution (0.0625 mg/l to 256 mg/l) was placed in four wells for each concentration. Twelve wells formerly incubated with bacteria were filled with 200 μl TSB only and served as positive controls; 12 wells with only TSB served as negative controls. After another 24 h in the brood chamber, the wells were emptied via deflection, washed with phosphate buffered saline (PBS), fixed with 150 μl 2.5% glutaraldehyde (Glutaraldehyde solution Grade II, 25%, Sigma–Aldrich®, St. Louis, MO, USA), diluted 1 to 10 in Aqua bidestillata for 15 min and dried at 60 ° C for 30 min. The dried biofilms were stained with 150 μl crystal violet (Merck KGaA®, Darmstadt, Germany) for 5 min and washed with PBS: 150 μl 33% acetic acid (AnalaR Normapur, Prolabo®, VWR International®, USA) was put in each well to improve optical density for quantification of biofilm with a BEP II-photometer (Zenyth 340st, Anthos Labtec BV®, Heerhugowaard, Netherlands). The optical density was measured at 405 nm, with the background measured at 620 nm. The MBC was considered to be the concentration of dalbavancin, which led to a 50% reduction in optical density.

## Results

**Fig. 1:**
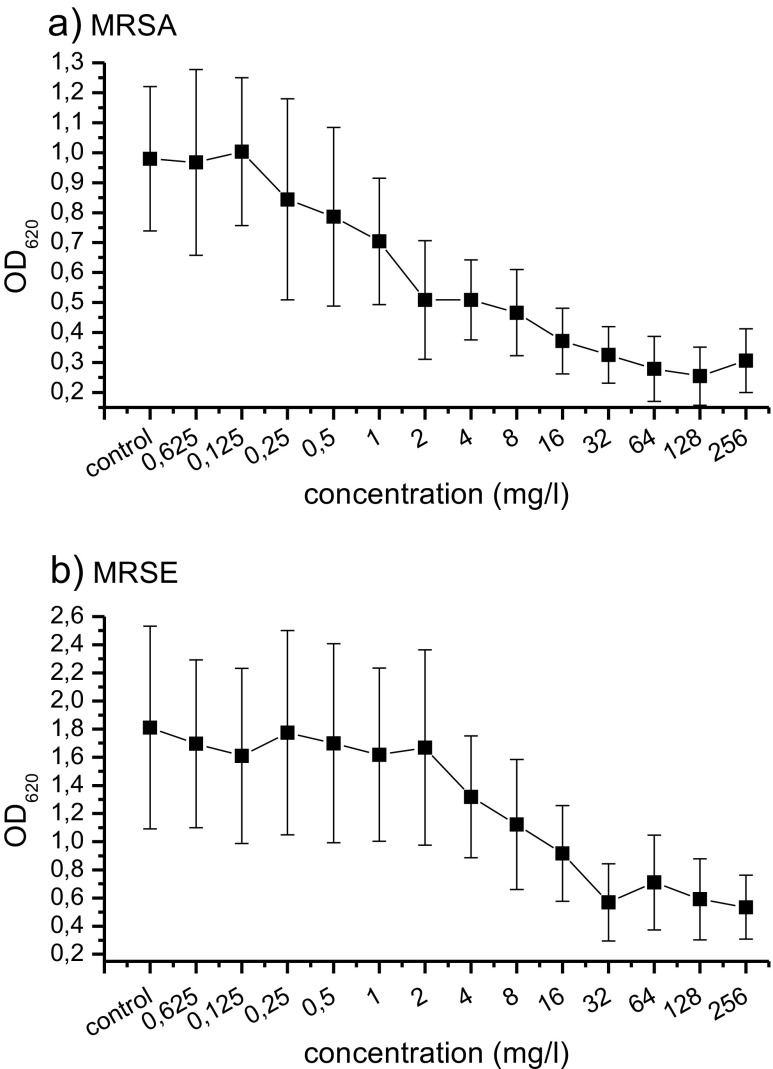
Mean OD_620_ for MRSA (μ_OD620_ = 0.579; SD _OD620_ = ± 0.186) and MRSE (μ _OD620_ = 0.952; SD _OD620_ = ± 0.318) in biofilms incubated with dalbavancin and TSB in decreasing concentrations measured with BEPII-Photometer

For planktonic cells, the MIC of dalbavancin ranged from 0.032 mg/l to 0.064 mg/l for MRSA and from 0.023 mg/l to 0.0625 mg/l for MRSE. For MRSA biofilms, the MBC of dalbavancin ranged from 1 mg/l to 4 mg/l, and from 2 mg/l to 16 mg/l for MRSE. (Table 1, Fig. 1)

**Table 1:** Comparison of MICs and MBCs for MRSA (μ _MBC_ = 1.80 mg/l; SD _MBC_ = ± 0.919 mg/l) and MRSE (μ _MBC_ = 12.2 mg/l; SD _MBC_ = ± 8.967 mg/l) bloodstream isolates

Isolate	MRSA		MRSE	
	MIC (mg/l)	MBC (mg/l)	MIC (mg/l)	MBC(mg/l)
1	0.064	4	0.023	8
2	0.064	2	0.0625	8
3	0.032	2	0.032	16
4	0.032	2	0.023	4
5	0.047	1	0.047	2
6	0.032	2	0.023	4
7	0.0312	1	0.0625	16
8	0.064	2	0.047	16
9	0.0312	1	0.032	16
10	0.0312	1	0.032	16

## Discussion

The aim of this study was to investigate the activity of dalbavancin against MRSA and MRSE growing in biofilms. The advantages of dalbavancin include its weekly dosing regimen and its high efficacy against gram-positive prokaryotes. In a study published by Raad et al., the efficacy of dalbavancin (one biweekly dose) compared with a 14-day course of vancomycin (twice a day) in patients with catheter-related bloodstream infection was evaluated [8]. The study showed dalbavancin to have a higher overall success rate compared to vancomycin. As a result it was hypothesized that due to the high plasma concentrations of dalbavancin, its penetration into biofilms could be significantly higher than that of vancomycin. However, until this point, in-vitro activity of dalbavancin against biofilms has not been evaluated [8]. From the results of this study, dalbavancin shows in-vitro activity against biofilms with MRSA and MRSE in concentrations between 1 and 16 mg/l, which are concentrations easily reached in vivo, as mean plasma concentrations have been shown to be > 35 mg/l for 7 days after one dose of 1,000 mg [6]. Another study conducted by Baldoni et al. investigated the activity of dalbavancin against planktonic and biofilm MRSA in a foreign-body infection model in guinea pigs. Dalbavancin led to a reduction of planktonic MRSA in cage fluid, but failed to eradicate biofilm MRSA from cages [7]. Only when dalbavancin was combined with rifampicin could reduction of biofilm MRSA be observed [7]. We suspect this finding to be due to higher protein binding in the in-vivo model, as the intraperitoneally applied concentrations of dalbavancin resemble the concentrations used in our in-vitro model [7]. Furthermore, metabolic processes after intraperitoneal application play a crucial role in the in-vivo model. Despite the advantages of subcutaneous animal foreign body models, they also have some limitations. In pharmacokinetic studies, differences in metabolic processes in small animals compared to humans have to be taken into account [11]. To further understand these findings, foreign body models with intravenous application of dalbavancin are needed. Focusing on in-vitro effects of dalbavancin, we observed previously undescribed properties of this antimicrobial agent. In the test runs performed prior to our experiment, we dissolved 500 mg of dalbavancin in 25 ml aqua bidestillata and 5% glucose (50 mg/ml) as described in the package information leaflet, and performed the same biofilm assay as described above; however, no reduction of biofilm could be observed. The OD_650_ was even higher in the wells filled with the highest dalbavancin concentrations, which made us revise our method. Comparing our results of dalbavancin in TSB with our pretests of dalbavancin in glucose, we concluded that in-vitro biofilm growth of MRSA and MRSE might be enhanced by glucose even when combined with dalbavancin. This effect is very likely to apply to the in-vitro model only, as glucose is metabolized in vivo after intravenous administration. We conclude that dalbavancin successfully reduced biofilms in vitro at concentrations which can be easily obtained in vivo, which substantiates the previously published findings that dalbavancin might have considerable potential as a drug in medical device infections, for which it is currently not approved [6]. In conclusion, because of the activity of dalbavancin on MRSA and MRSE biofilms demonstrated in this study, we recommend further studies on the activity and efficacy of dalbavancin in in-vivo models with medical device associated infections, as a biweekly dosing regimen could not only reduce healthcare costs, but also improve patients’ quality of life.

## References

[1] Hall-Stoodley L, Costerton JW, Stoodley P (2004). Bacterial biofilms: from the natural environment to infectious diseases. Nat Rev Microbiol.

[2] Donlan RM (2001). Biofilms and device-associated infections. Emerg Infect Dis.

[3] Parsek MR, Singh PK (2003). Bacterial biofilms: an emerging link to disease pathogenesis. Annu Rev Microbiol.

[4] Simor AE, Pelude L, Golding G (2016). Determinants of outcome in hospitalized patients with methicillin-resistant *Staphylococcus aureus* bloodstream infection: results from national surveillance in Canada, 2008–2012. Infect Control Hosp Epidemiol.

[5] Zajonz D, Wuthe L, Rodloff AC et al (2015) Infections of hip and knee endoprotheses: spectrum of pathogens and the role of multiresistant bacteria. Chirurg 87(4):332–339 [Article in German] 10.1007/s00104-015-0126-5 26661951

[6] Seltzer E, Dorr MB, Goldstein BP (2003). Once-weekly dalbavancin versus standard-of-care antimicrobial regimens for treatment of skin and soft-tissue infections. Clin Infect Dis.

[7] Baldoni D, Furustrand Tafin U, Aeppli S (2013). Activity of dalbavancin, alone and in combination with rifampicin, against methicillin-resistant *Staphylococcus Aureus* in a foreign-body infection model. Int J Antimicrob Agents.

[8] Raad I, Darouiche R, Vazquez J (2005). Efficacy and safety of weekly dalbavancin therapy for catheter-related bloodstream infection caused by gram-positive pathogens. Clin Infect Dis.

[9] Streit JM, Fritsche TR, Sader HS (2004). Worldwide assessment of dalbavancin activity and spectrum against over 6,000 clinical isolates. Diagn Microbiol Infect.

[10] Christensen GD, Simpson WA, Bisno AL (1982). Adherence of slime-producing strains of *Staphylococcus Epidermidis* to smooth surfaces. Infect Immun.

[11] Nowakowska J, Landmann R, Khanna N (2014). Foreign body infection models to study host-pathogen response and antimicrobial tolerance of bacterial biofilm. Antibiotics.

